# The Penna Model of Biological Aging

**Published:** 2009-11-24

**Authors:** D. Stauffer

**Affiliations:** Institute for Theoretical Physics, Cologne University, D-50923 Köln, Euroland

**Keywords:** Monte Carlo simulations, life, death, reproduction

## Abstract

This review deals with computer simulation of biological aging, particularly with the Penna model of 1995. They are based on the mutation accumulation theory of half a century ago. The results agree well with demographical reality, and also with the seemingly contradictory influence of predators on the aging of prey.

## Introduction

“There is no shortage of theories of ageing” (Partridge, 2007). Indeed it has been claimed that there as many aging theories as there are aging theorists. This claim is inaccurate since this author alone simulated three different types of aging theories.

However, for this journal we are interested in computer simulations of biological aging, and then the number of candidates goes down drastically. I do not claim that only those theories which have been simulated extensively are correct, and that the other theories are wrong; I merely report on what readers of this journal presumably are interested in. Thus in the following chapters I review recent progress on the most widespread model of biological aging, which was invented by Thadeu Penna before he was 30 years old (Penna, 1995). At the end, other models will be reviewed more briefly. There are many review articles and two books (Moss de Oliveira et al. 1999; Stauffer et al. 2006) on this subject; thus we summarise here the research with emphasis on recent developments.

## Reality

Every year we age by one year if we do not die, and in middle age the mortality increases roughly exponentially with age *a*,

(1a) $$\text{mortality}\propto e^{ba},$$

as observed by Gompertz in 1825. This mortality can be defined as

(1b) q=[S(a)−S(a+1)]/S(a)

where *a* is the age (usually measured in years for humans) and *S*(*a*) the number of people surviving from birth to age *a*; this *q* is the fraction of people who reach age *a* and die before they reach age *a* + 1. The Gompertz law describes reality better if instead of this *q* one looks at the mortality function (or force of mortality)

(1c) μ=−dln(S(a))/da

since this age derivative is not based on one year as the only relevant time interval and can become larger than one. If only life-tables for *S*(*a*) based on years are available, one may approximate this mortality function as

(1d) μ≃ln[S(a)/S(a+1)]

Both expressions for *μ* mathematically can go to infinity for old age, as required by the Gompertz law, while *q* by definition cannot exceed unity.

**Figure 1.**
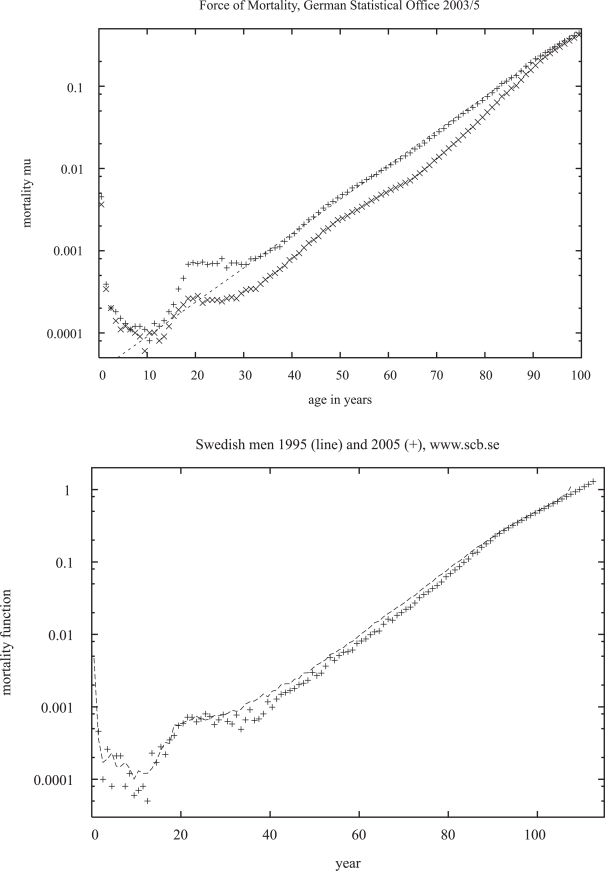
Mortality function *μ* versus age for: a) German men (+) and women (×); b) Swedish men.

Many people want to become young again, and when for some flies a mortality plateau was seen at very old age, claims were made that also for humans the mortality has a maximum somewhat below 100 years. However, the facts of Figure 1 are less optimistic, and only above 110 years human mortality might reach a constant (Robine and Vaupel, 2001). For the oldest Swedish men the mortality function *μ* in Figure 1 is above 1, invalidating the earlier and then very good fit (Thatcher et al. 1998)

$$\mu =e^{ba}/[\text{const}+e^{ba}]$$

The downward deviations from the simple exponential increase of the Gompertz law (1a) may become the smaller the better the data are (Gavrilova and Gavrilov, 2005).

Women should not be relied upon in tests of this exponential mortality increase. While men obey it quite nicely in Figure 1, the lawless women first have a mortality only about half as large as men, then increase their mortality stronger than the men until finally the two mortalities become about equal near the age of 100 years. Thus if one plots female mortalities only above the age of 80 one sees strong curvature which can be misinterpreted as a mortality deceleration away from the Gompertz law (1a).

**Figure 2.**
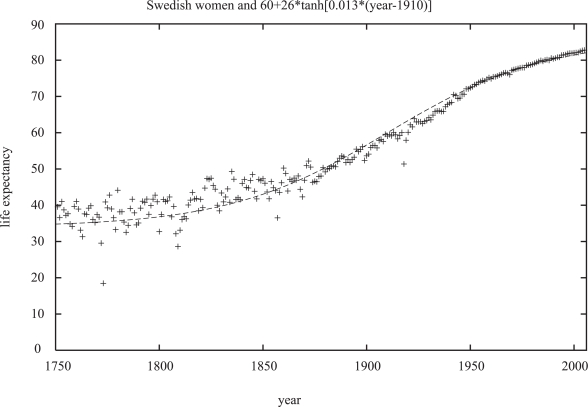
Life expectancy at birth for Swedish women over 2.5 centuries, also showing the improved statistical accuracy after the creation of a statistical central bureau in Sweden around 1860, The influenza pandemic in seen in 1918.

Germans also should not be relied upon. Wars may have long-time consequences, and German life expectancies in the 1960’s decreased for a few years instead of the usual increase. The unification of East and West Germany in 1990 lead to womankind’s greatest birth strike in East Germany (now mostly over), with the number of children per women falling below one for some years. Sweden, in contrast, avoided wars since two centuries, and Swedish life expectancy at birth also increased smoothly but non-linearly since two centuries, Figure 2, without showing a reliable tendency to become constant.

If one checks for each year in which country the life expectancy at birth is highest, and then plots this life expectancy versus the calendar year, one sees roughly a straight line (Oeppen and Vaupel, 2002) since 160 years, with Japan leading since many years (Cheung and Robine, 2007). This linerarity was hardly valid around the year 1800, Figure 2.

If one compares different countries and different centuries, one sees some universality, going back to Mildvan and Strehler but formulated in proper dimensionless form by Azbel 1996 (see also Gavrilov and Gavrilova, 2001):

(2) μ=const b exp [b(a−X)]

where *X* ≃ 103 years is a characteristic age (not a maximum age) for all human societies, independent of country and calendar years. Medical progress in the last two centuries increased the Gompertz slope *b* but not the characteristic age *X*. However, during the last few decades one may have seen a change to a constant b and an increasing *X* (Wilmoth et al. 1999 and 2000; Yashin et al. 2001; Edwards and Tuljapurkar, 2005; Cheung and Robine, 2007).

Neither the latter changes not the downward deviations from the Gompertz law are well established, and thus eq(2) is a good approximation for humans above the age of 30 years.

At young age, drastic deviations are seen in Figure 1 from the straight line symbolizing the Gompertz law (1a). Adding a constant to eq(1a) may fit between 20 and 30 years, but still fails below 20 years. Mostly we will thus ignore child mortality and try to simulate models for adult mortality: Don’t trust anybody below 30.

In all our discussions, “aging” is defined through the mortality, not through wisdom, beauty, health etc. Not only are mortalities much better documented and measurable than wisdom, they are also best suited for computer simulations through population dynamics. A good aging model thus should reproduce the empirical Gompertz law (1a), i.e. the exponential increase of adult mortalities with age. Refinements then should explain the mortality minimum at childhood, and also the difference between male and female mortalities except for very old people. Once that is achieved one may apply the model for other questions like speciation or demography.

## Mutation Accumulation and Penna Model

More than half a century ago, Medawar tried to explain aging through the accumulation of inherited bad mutations over many generation. If such a hereditary disease kills a person at young age (before sexual maturity), that person has not produced any children, and this mutation will fail in Darwinian survival of the fittest. If, one the other hand, such a bad mutation kills this author at about the time this review is printed, then the government can save pension money. Thus bad mutations acting at young age can hardly spread in the population due to Darwinian selection pressure; for mutations acting at old age this pressure is much weaker, and these mutations can spread widely. This argument is not restricted to humans and indeed the Gompertz law also applies to many animals (Vaupel et al. 1998), with different time scales for the slope *b* and the characteristic age *X*.

Penna implemented this mutation accumulation hypothesis by dividing life into 32 time intervals and by representing the genome (DNA) through a string of 32 bits, each of which can be zero or one. A zero bit means health, a bit set to one means a dangerous inherited disease starts to act from that age on which corresponds to the position of this bit in the bit-string. If *T* (typically, *T* = 3) bits are active, their combined effect kills the individual. Each individual which has reached the minimum reproduction age of *R* (typically, *R* = 8) gets *B* (typically, 1 ≤ *B* ≤ 4) children at each time step, where 32 time steps give the maximum life span. The child inherits the mother’s genome except for *M* (typically, *M* = 1) mutations of randomly selected bits where a zero bit becomes a one bit. (A bit set to one remains at one in most versions of this model.) The number 32 is computationally convenient but biologically unrealistic; longer bit-strings are better and give roughly the same results if parameters are scaled properly (Łaskiewicz et al. 2005). In any case, parameters should be chosen such that in the stationary equilibrium no individual reaches the age of 32 (or whatever the length of the bit-string is), since there it would die because of computational convenience and not for biological reasons.

**Figure 3.**
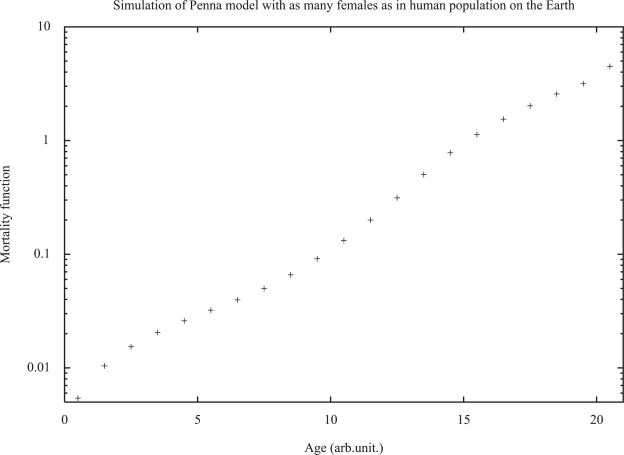
Simulated mortality function for the asexual Penna model with 32 bits and *R* = 8. From Stauffer et al. (2006).

Starting with an ideal set of zero bits for all individuals, after about 10^3^ iterations the total population achieves a stationary value, and after about 10^4^ iterations this is true also for the oldest individuals. Figure 3 shows large-scale simulations in reasonable agreement with the Gompertz law. Results do not strongly depend on the various parameters. However, it may happen that for too low birth rates the whole population dies out (Malarz, 2007); this effect was also studied for a host-parasite system (Stauffer et al. 2007). For the oldest old the mortality may jump to infinity but this is hardly seen in simulations.

The distribution of bits set to one shows a low probability for young age, though *T* – 1 randomly selected bit positions may have most bits set to one for the whole population. Then, after the minimum reproduction age *R*, the fraction of mutated bits rises first slowly, then sharply, and reaches unity at some well-defined age which is about the maximum age in that population. For shorter times like 10^3^ iterations this maximum age is not yet sharply defined (Sitarz and Maksymowicz, 2005).

## Applications of Asexual Model

To avoid a population growing exponentially to infinity, a Verhulst death probability *N*(*t*)/*K* is applied at each time step *t*, where *N* is the current total population and *K* is often called the carrying capacity, describing the limits of food and space. These deaths can be applied to everybody or (computationally more problematic but biologically more realistic) to newborn babies only (Martins and Cebrat, 2000). The latter choice is implemented indirectly if everybody is put on a lattice and offspring have to be placed on an empty lattice neighbor; then no Verhulst deaths are needed at all.

Life means to eat and to be eaten. He et al. (2005) put wolves, sheep and grass onto a square lattice and let all animals age according to the Penna model. They found strong oscillations as is often the case in such prey-predator models, but also possible extinction of the wolves and even of the sheep.

One biological observation regarded as crucial support for the mutation accumulation hypothesis are the Virginia opossum. These small mammals have a low reproductive age and a low life expectation on the continent where they are hunted by predators. But on islands without such predators, they get offspring later and die later. Such “plasticity” of the minimum age of reproduction is consistent with mutation accumulation but difficult to explain otherwise. Recently, Reznick et al. (2004) found the opposite effect for other animals. Fortunately, Altevolmer (1999) in between these biological observations simulated this plasticity in the Penna aging model and found both decreases and increases of the minimum age of reproduction, depending on whether the predators kill mainly the young or mainly the old prey. Thus simulations predicted some observations, and these observations do not contradict the mutation accumulation hypothesis in the Penna implementation. (Mitteldorf and Pepper, 2007 showed what difficulties arise if one ignores this Penna model research.)

If everybody in the present model has the same genome, then their genetic death ages all agree, and the only variation would come from the Verhulst deaths (if these are not restricted to newborns). Biological experiments are sometimes made with inbred populations believed to have the same genes, but nevertheless not all individuals die at the same age. This bad property of the present model was repaired by Pletcher and Neuhauser (2000) by combining it with reliability theory (Gavrilov and Gavrilova, 2001). Alternatively one may assume that too many active mutations do not kill automatically, but only with a probability depending on the difference of the active number of mutations and the threshold *T* (Coe et al. 2002).

## Sexual Penna Model

Bacteria pretend not to have sex but sometimes they engage in exchange of genome. (“parasex”). More complicated organisms often divide into male and female individuals, and off-spring is created by combining parts of the paternal genome with parts of the maternal genome, using recombination. We ignore some complications of reality and represent the paternal genome in a sperm cell by one bit-string, and the maternal genome in the egg cell by another bit-string. The child gets both bit-strings and thus has a diploid genome with two bit-strings of the same length. In reality these two bit-strings correspond to two chromosomes, not to two nucleotide sequences on the DNA double helix (Bernardes 1996, for a long review of early research see Moss de Oliveira et al. 1999).

To create the paternal bit-string in the haploid sperm cell, the two paternal bit-strings undergo with some probability (often taken as unity) a crossover (recombination): Some position on the bit-string is selected randomly, and all the bits from on one side of this crossover point are taken from one of the two bit-strings, and all the other bits from the other bit-string. In this way, the sperm cell has a bit-string different from each of the two paternal bit-strings. An analogous random crossover creates the haploid genome of the egg cell. Then these two bit-strings fuse to form the diploid zygote from which develops into the child. The resulting greater variety is supposed to justify the cost of sexual reproduction compared to bacterial cloning but it is not clear that the profit outweighs this cost (Martins and Stauffer, 2001): The city of Cologne later imposed a sex tax. Scharf (as cited on page 91 of Stauffer et al. 2006) offered the additional argument of pre-selection: Only the fastest sperm cell can fertilize the egg cell, and cells with bad mutations may move slower and thus are less likely to enter the egg cell. This Darwinian preselection of the fittest sperm cell does not exist for asexual reproduction and may justify the existence of men. Fortunately for me, Mother Nature invented too late the trick to produce males without mouth and stomach, suited only as fertilization machines with a high profit-to-cost ratio.

All men know that they die sooner than women because they are oppressed by them. Scientific journals, however, feel obliged to publish also other reasons. One blames Mother Nature: Women have two X chromosomes, and men have one X and one Y chromosome, with the Y chromosome containing only few genes. Using a sexual Penna model with many chromosomes, one of them the X (or Y), Schneider et al. (1998) found male mortalities to be about twice as high as female mortalities, except for the oldest old where the two roughly agree.

A crucial test of this chromosome hypothesis would be life expectancies for birds. While for mammals the males have different (XY) and the females the same (XX) chromosomes, for birds the situation is the opposite. Unfortunately two studies (Paevskii, 1985; Austad, 2001) contradict each other and no better ones are known to me. For humans the male-female differences in life expectancies are now usually several years (Oeppen and Vaupel, 2002) and may be larger than the gains if cancer can be healed; thus understanding these differences could be very helpful for human health. However, breeding, feeding, protecting and studying many thousand birds up to their intrinsic deaths seems not be to be as sexy as analyzing human genomes or claiming to heal Alzheimer by stem cell research.

**Figure 4.**
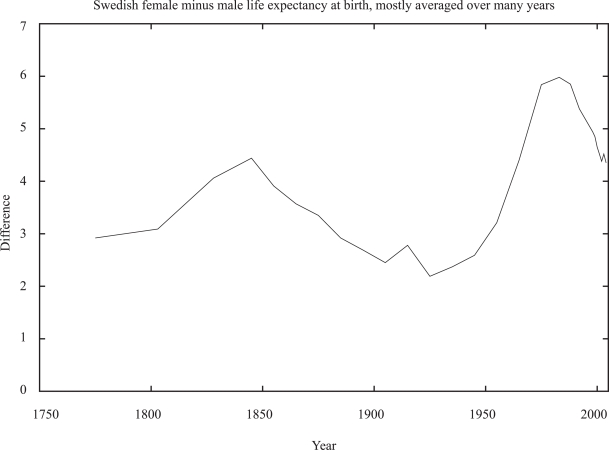
Time variation of the advantages in life expectancies of women over men in Sweden. From Stauffer et al. (2006) and www.scb.se.

In any case, genetics alone can hardly explain *all* the differences in the life expectancies of men and women, since in Sweden this difference changed rapidly over 250 years, Figure 4, too short a time to change appreciably the human genome; Glei and Horiochi (2007) surveyed more completely many rich countries over many decades.

An important result of these sexual Penna model simulations is that menopause or its analogs, that means the cessation of female reproductive ability long before death, can emerge by itself as a result of the need for child care by the mother and an increase with age in the risk of giving birth. There is no need of human culture and grandmothers teaching their grand children to explain menopause, and indeed similar effects have been observed in many animals, not just in pilot whales as was thought long ago. Stauffer et al. (2006) discuss this point at length.

Sexual, as opposed to asexual, reproduction allows a new way to escape the detrimental effects of bad mutations. If we have two bit-strings of length 8 in the asexual case then 00010000 is clearly more favorable for survival than 11101111. However, for sexual reproduction most mutations are recessive, i.e. the reduce the survival probability if and only if both bits are set at the same place in the two bit-strings. If these two bit-string examples form the diploid genome, and if all bit-positions are recessive, then the survival probability is not reduced at all, in spite of half the bits being set to one. Thus survival of the fittest may lead to individuals which have half their bits set to one but with complimentary bit-strings in the two bit-strings of the haploid genomes.

**Figure 5.**
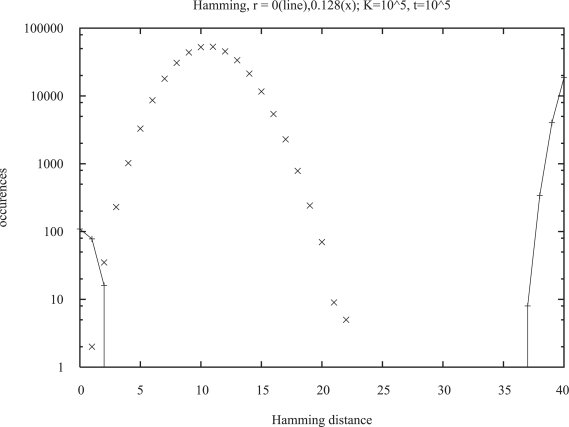
Distribution of Hamming distances between the two bit-strings of each diploid genome with low (line) and intermediate (×) recombination probabilities *r*. From Bo kowska et al. 2007, see also Cebrat and Stauffer 2008.

This possibility was actually realized in models both from a genetics group (Zawierta et al. 2007; Waga et al. 2007; Cebrat and Stauffer, 2008) and from physicists (Pękalski 2007b; de Oliveira et al. 2007) without aging. It also appears in the Penna model, if the recombination rate (crossover probability) is not equal to one as traditionally but is much smaller. We can measure this effect more quantitatively by the Hamming distance between the two bit-strings of the same individual. This Hamming distance counts how many bits are different in a position-by-position comparison of the two bit-strings. We compare only the bits up to the minimum reproduction age *R*, since those at higher ages are mostly set to one anyhow. Figure 5 shows for bit-strings of length 64 and *R* = 40 the normal behavior (x) at a rather high recombination probability *r* = 0.128; there is a single peak with a maximum at the rather low value of ten (of 40) bits: Evolution tried to reduce the number of mutations. At *r* = 0, on the other hand, the distribution splits up into two parts, one near the maximum of 40 and one near zero. In most of the population one finds only two different bit-strings which are complementary to each other. And most individuals have these two complimentary bit-strings in their diploid genome: Evolution pushed for complimentary mutations at small *r*, and for elimination of mutations at large *r*. These effects remain also if gamete recognition selects for the formation of a zygote only those bit-strings which differ on their first bit. Waga et al. (2007) used such concepts to simulate sympatric speciation, but without the Penna aging model.

Such speciation means that one species slowly separates into two different ones, without being separated by a geographical barrier. (Allopatric speciation is caused by such a barrier, like *homo sapiens coloniensis* and *homo sapiens neanderthalensis* being today separated by the Rhine river.) Luz-Burgoa et al. (2006) expanded the Penna aging model by giving each individual besides the usual two-bit-string another pair of bit-strings, which are not related to aging. Roughly, the number of bits set to one is called the phenotype. The Verhulst death probabilities depend on the phenotype, and so does the mating preference: females prefer mating partners with the largest (smallest) phenotype, if their own phenotype is larger(smaller) than average. Figure 1 of Luz-Burgoa et al. shows how an initial single-peaked distribution of intermediate phenotypes divided after many iterations into two widely separated peaks at the largest and smallest possible phenotypes: Two species emerged out of one, without any outside influence from two different food sources etc. (See Pękalski, 2007a for a simplified model)

Practical applications include demography: How many young people have to support the upcoming retirement of this author? Bońkowska et al. (2006) simulated this with a sexual Penna model, where each bit-string has a length of 640 bits. Much simpler are the simulations (sections 6.1 and 9.5 in Stauffer et al. 2006; Sumour et al. 2007) where one age distribution of people is iterated to give the age distribution in the next year, using realistic birth rates and Gompertz mortalities. Many European countries may need young immigrants and increased retirement ages to deal with the reduced birth rates and longer life expectancies of recent decades.

## Other Aging Simulations

Weismann at the end of the 19th century suggested roughly that we die to make place for our children. In some sense this idea is implemented by a simple model (Stauffer and Radomski, 2001) which avoided all bit-string complications and had only two genetic properties for each individual: the minimum reproduction age *R* and the genetic death age *D*. Both mutated randomly up or down from one generation to the next. Without further restrictions the death age goes to infinity, nice but unrealistic. But if the birth rate was assumed to be roughly inversely proportional to *D* − *R*, such that the expected number of offspring is constant and no longer influenced by mutations in *R* and *D*, a stable equilibrium was obtained: A finite death age is advantageous for the population as a whole, in agreement with Weissmann. Modifications are needed to get a mortality increasing exponentially instead of linearly with age (Makowiec et al. 2001). While several publications were devoted to this model, its agreement with reality was not as easy to obtain as for the Penna model.

Also quite simple are the models of Partridge and Barton, Jan, Dasgupta, Heumann and Hötzel with two self-organizing mortalities, for juveniles and adults. They finally lead to rather realistic mortalities for many ages (Medeiros and Onody, 2001). Later this model was used by Lobo and Onody (2006) to explain sex. Continuation by others of this work by the Onody group might be promising.

A reasonable mortality function was also obtained in a telomere model by Masa et al. (2006). The simplest and most recent model by Shklovskii (2005) does not even require a computer simulation and uses reliability theory (Gavrilov and Gavrilova, 2001) for the immune system.

All these quantitative models, plus many earlier theories reviewed by Kirkwood (2005) have not reached the popularity of the Penna model. With respect to practical applications like rejuvenation pills, not much progress has been made since the experiment of Grant et al. (1952).

## Conclusion

The Penna model of 1995 is the most widespread computer simulation of aging by mutation accumulation, since it gave from the beginning in a simple way the roughly exponential increase of mortality with age for adult humans. Had the alternative of Medeiros and Onody (2001) been published immediately after the 1993/5 models of Partridge and Barton, Jan, Dasgupta, Heumann and Hötzel on which it is based, research might have taken a different path.

There are many other explanations of aging besides mutation accumulation (Kirkwood, 2005) but they are not as suitable for computer simulation. This does not mean that they are wrong. Possible, many of them are correct and describe one of the many aspects of aging. There may exist a human longevity gene; oxygen radicals may destroy our DNA; …. It is not even clear that the various theories contradict each other. A longevity gene may enhance the protection against oxygen radicals and thus reduce the danger of bad mutations on which mutation accumulation is based; thus all three theories work together.

I thank all my collaborators in aging research, in particular N. Jan, T.J.P. Penna, S. Moss de Oliveira, P.M.C. de Oliveira, A.T. Bernardes, S. Cebrat, D. Makowiec, A. Maksymowicz, and A.O. Sousa (in temporal order).
